# Research for new drugs for elimination of onchocerciasis in Africa

**DOI:** 10.1016/j.ijpddr.2016.04.002

**Published:** 2016-05-19

**Authors:** Annette C. Kuesel

**Affiliations:** UNICEF/UNDP/World Bank/WHO Special Programme for Research and Training in Tropical Diseases, 20 Avenue Appia, 1211 Geneva, Switzerland

**Keywords:** Onchocerciasis, Elimination, Africa, Discovery, Drug development, Implementation research

## Abstract

Onchocerciasis is a parasitic, vector borne disease caused by the filarial nematode *Onchocerca volvulus*. More than 99% of the population at risk of infection live in Africa. Onchocerciasis control was initiated in West Africa in 1974 with vector control, later complemented by ivermectin mass drug administration and in the other African endemic countries in 1995 with annual community directed treatment with ivermectin (CDTI.) This has significantly reduced infection prevalence. Together with proof-of-concept for onchocerciasis elimination with annual CDTI from foci in Senegal and Mali, this has resulted in targeting onchocerciasis elimination in selected African countries by 2020 and in 80% of African countries by 2025. The challenges for meeting these targets include the number of endemic countries where conflict has delayed or interrupted control programmes, cross-border foci, potential emergence of parasite strains with low susceptibility to ivermectin and co-endemicity of loiasis, another parasitic vector borne disease, which slows down or prohibits CDTI implementation. Some of these challenges could be addressed with new drugs or drug combinations with a higher effect on *Onchocerca volvulus* than ivermectin. This paper reviews the path from discovery of new compounds to their qualification for large scale use and the support regulatory authorities provide for development of drugs for neglected tropical diseases. The status of research for new drugs or treatment regimens for onchocerciasis along the path to regulatory approval and qualification for large scale use is reviewed. This research includes new regimens and combinations of ivermectin and albendazole, antibiotics targeting the *O. volvulus* endosymbiont *Wolbachia*, flubendazole, moxidectin and emodepside and discovery of new compounds.

## Highlights

• Challenges for onchocerciasis elimination requiring new drugs.
• Summary of path from discovery to qualification of drugs for human use.
• Regulatory support and incentives for development of drugs for neglected diseases.
• Development of new dose regimens, drug combinations and new drugs against *O. volvulus*.

## 1 Introduction

### 1.1 Onchocerciasis

**Figure:**
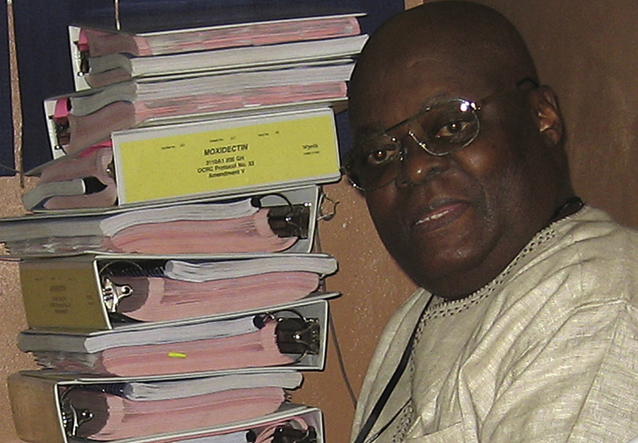

**Dr. Awadzi (13.06.1939-16.03.2011)**

Father of ethical, systematic and evidence based clinical research in onchocerciasis Dr. Awadzi was instrumental in development of the methods for assessing the efficacy and safety of anti-onchocercal drugs, providing training and collaboration for many young scientists and physicians. He evaluated nearly all drug candidates for onchocerciasis proposed before and after the development of ivermectin. This picture shows him with the case report forms of the last clinical trial he lead as principal investigator, the Phase 2 study of moxidectin. He then ‘retired’ and was the scientific advisor on the Phase 3 study and trained, together with his collaborators at the Onchocerciasis Chemotherapy Research Center, Hohoe, Ghana (now University of Health and Allied Sciences Research Centre (UHASRC), School of Public Health, Ghana) the principal investigators of that study in Liberia and the Democratic Republic of the Congo.

Onchocerciasis (river blindness) is a parasitic, vector borne disease caused by the filarial nematode *Onchocerca volvulus*. Four life stages of *Onchocerca volvulus* live in humans: the infective (L3) larvae injected by the vector undergo two moultings to develop via L4 larvae into juvenile adults (L5) and mature to reproductively competent adults (macrofilariae) within around 1 year (Duke, 1991, Basanez and Ricardez-Esquinca, 2001). The macrofilariae (adult worms) have an estimated mean reproductive life span of 9–11 years (Plaisier et al., 1991), reside primarily in subcutaneous and deep tissue nodules and produce embryos (microfilariae). The microfilariae live for around 1 year, reside primarily in the subepidermal layer of the dermis and can invade the eyes. The immunological response to the death of the microfilariae, which includes inflammatory components, is responsible for the symptoms of the disease which range from itching to blindness. The vectors are species of the genus *Simulium* (black flies), in Africa primarily *Simulium damnosum* s.l.. During a blood meal, the blackflies ingest microfilariae present in the skin which develop into infective (L3) larvae. Transmission of the infective larvae to humans upon another blood meal closes the transmission cycle (World Health Organization, 1995).

### 1.2 Onchocerciasis control programmes

The significant public health and resulting socio-economic burden of onchocerciasis have motivated large scale disease control and elimination programmes in all endemic countries. The vast majority of these countries are in Africa, where more than 100 Million people live in onchocerciasis endemic areas (Noma et al., 2014, Zoure et al., 2014, O’Hanlon et al., 2016). Around 0.56 Million people were estimated to live in the 13 small endemic foci in 6 countries in Central and South America (Center for Disease Control (CDC), 2013) and around 0.3 Million in Yemen (Mackenzie et al., 2012) Onchocerciasis control started in 1974 with the Onchocerciasis Control Programme in West Africa (OCP) which conducted large scale larviciding of vector breeding sites in the West African Savannah. The vector-control based strategy was complemented with mass drug administration (MDA) (Boatin and Richards, Jr. 2006) after ivermectin (Mectizan^®^) had been registered and Merck decided to donate ivermectin for onchocerciasis control for as long and in the amounts needed (Campbell, 2012). At the closure of OCP in 2002, onchocerciasis had been eliminated as a public health problem in the programme area (Benton et al., 1998, Boatin, 2008, Campbell, 2012).

Mass ivermectin administration was the sole strategy in the countries of the Onchocerciasis Elimination Program for the Americas (OEPA). The public health system distributed ivermectin initially annually, then twice yearly in all villages in which onchocerciasis was endemic. This was complemented for several years by two additional distributions in the majority of hyper-endemic communities (≥60% prevalence before the start of interventions) and a few meso-endemic communities (≥20% to <60% prevalence before start of interventions). In all but the large focus spanning areas of Venezuela and Brazil, this strategy has or is likely to have permanently interrupted transmission of the parasite (Convit et al., 2013, Anonymous, 2013, West et al., 2013, Center for Disease Control (CDC), 2013, Lovato et al., 2014, Rodriguez-Perez et al., 2015, Anonymous, 2015).

In Yemen, ivermectin has been offered since 1992 at least biannually to those with symptoms (Al-Qubati, 1994, Al-Qubati et al., 1997). Yemen is targeting onchocerciasis elimination through combination of ivermectin mass drug administration and vector control (World Health Organization, 2012a).

In 1995, the African Programme for Onchocerciasis Control (APOC) was launched in view of eliminating onchocerciasis as a public health problem in the African countries not covered by the OCP (Roungou et al., 2015, Fobi et al., 2015). The strategy was annual community-directed treatment with ivermectin (CDTI) in all communities where onchocerciasis was meso- or hyper-endemic (Noma et al., 2014). In 2014, around 112 Million people received ivermectin in the 22 countries which reported data to APOC. Treatment data from another 4 countries implementing CDTI had not been reported to APOC (African Programme for Onchocerciasis Control (APOC) 2015b). Field-based as well as modelling studies of the impact of annual CDTI show that in areas with long term CDTI, onchocerciasis has indeed been eliminated as a public health problem (Ozoh et al., 2011, Coffeng et al., 2013, Coffeng et al., 2014b, Turner et al., 2014).

### 1.3 Ivermectin - the ’standard of control’

Ivermectin exerts a ’microfilaricidal’ effect which results in near elimination of microfilariae from the skin within a few days and a so called ’embryostatic’ effect which temporarily prevents release of microfilariae by the macrofilariae (Basanez et al., 2008). Together, these effects result in long term reduction of microfilariae levels in infected people. The speed and extent of reduction of skin microfilariae levels as well as the time and extent of repopulation of the skin with microfilariae vary between people (Churcher et al., 2009, Awadzi et al., 2014). Time and rate of repopulation of the skin have been linked to host factors, including the immune competence (Ali et al., 2002) and the number of palpable nodules, a ’proxy’ for the number of macrofilariae (Pion et al., 2011). Reduction of microfilariae levels prevents appearance of new symptoms, eliminates reversible symptoms of the disease, prevents progression of irreversible symptoms and reduces parasite transmission.

Ivermectin is suitable for MDA because it has an excellent safety profile: adverse reactions are due to the efficacy of the drug, i.e. the immunological reaction of the body to the death of the microfilariae which includes inflammatory components (Mackenzie et al., 2003). These are referred to as ’Mazzotti reactions’ and are in most cases mild to moderate and disappear within a few days without treatment (Awadzi, 1980, DeSole et al., 1989a, DeSole et al., 1989b, Awadzi et al., 1990) even at doses higher than the one used in onchocerciasis control programmes (150 μg/kg) (Awadzi et al., 1999).

Severe adverse reactions to ivermectin treatment different from Mazzotti reactions can, however, occur in people heavily infected with the filarial parasite *Loa loa*. These reactions can have sequelae, be live-threatening and have even led to death. The risk of severe adverse reactions increases with the intensity of infection as measured by the microfilarial load. People in whom this load exceeds 30,000 microfilariae/ml blood are at particular risk of reactions that result in at least several days of incapacity to conduct normal everyday activities without assistance (Gardon et al., 1997, Boussinesq et al., 1998, Boussinesq et al., 2003, Scientific Working Group on Serious Adverse Events in Loa Loa endemic areas, 2003, Twum-Danso and Meredith, 2003, Twum-Danso, 2003a, Twum-Danso, 2003b). *Loa loa* is endemic in the equatorial forest areas of Central and West Africa. Its distribution has been mapped using a rapid assessment method (Zoure et al., 2011). In *Loa loa* endemic areas where onchocerciasis is meso- or hyperendemic, the benefit of ivermectin treatment is considered to exceed the risk of severe adverse reactions provided CDTI is implemented with special precautions to ensure that appropriate care is available for people developing severe adverse reactions. In contrast, in *Loa loa* endemic areas where onchocerciasis is hypoendemic, CDTI cannot be implemented at all and treatment with ivermectin needs to be individual, clinic-based (Mectizan Expert Committee and APOC Technical Consultative Committee, 2004).

The severe adverse reactions to ivermectin in people highly infected with *Loa loa* also delay progress towards elimination of lymphatic filariasis (LF) in Africa where the ’standard of control’ is co-administration of ivermectin and albendazole. People with LF do not directly benefit from ivermectin treatment since the symptoms of LF are due to the macrofilariae. Consequently, use of ivermectin in areas co-endemic for LF and *Loa loa*, but not at least mesoendemic for onchocerciasis, does not have an acceptable risk-benefit ratio. The provisional strategy for LF control in *Loa loa* co-endemic areas is twice yearly treatment with 400 mg albendazole complemented with vector control (World Health Organization, 2012b). This strategy is now supported by the results of a community trial of semiannual MDA with 400 mg albendazole. The study showed that the geometric mean microfilariae level 12 months after the first treatment was reduced by 60% relative to the pre-treatment level (Pion et al., 2015). Co-infections with other filariae do not appear to significantly affect the adverse reaction profile of ivermectin, presumably because parasite numbers are low relative to those in heavy *Loa loa* infections.

### 1.4 Moving towards onchocerciasis elimination in Africa

APOC was formed to support onchocerciasis endemic countries in establishing sustainable CDTI for control of onchocerciasis as a public health problem. At the time, it was considered impossible to eliminate onchocerciasis in Africa with CDTI alone (Dadzie et al., 2003).

Since then, a study in two onchocerciasis hyperendemic foci in Mali and Senegal has provided proof-of-concept that long term annual CDTI can eliminate onchocerciasis infection and permanently interrupt parasite transmission in Africa (Diawara et al., 2009, Traore et al., 2012). Encouraged by these results, APOC conducted large scale evaluation of the prevalence of infection and intensity of microfilaridermia in areas under long term CDTI. In close to 90% of the areas evaluated, prevalence of infection had decreased to or even below the level predicted by the onchocerciasis transmission model ONCHOSIM (Plaisier et al., 1990, Remme et al., 1990) when the pre-CDTI endemicity, the percentage of the population which participated in CDTI and the number of years of CDTI were taken into account (Plaisier et al., 1990, African Programme for Onchocerciasis Control (APOC), 2009, African Programme for Onchocerciasis Control (APOC), 2010a, African Programme for Onchocerciasis Control (APOC), 2011, Tekle et al., 2012, African Programme for Onchocerciasis Control (APOC), 2012a, African Programme for Onchocerciasis Control (APOC), 2013, African Programme for Onchocerciasis Control (APOC), 2014, Stolk et al., 2015, African Programme for Onchocerciasis Control (APOC), 2015a, African Programme for Onchocerciasis Control (APOC), 2015b. In the isolated meso-endemic focus Abu Hamed in the North of Sudan, annual CDTI initiated in 1998 and biannual CDTI from 2006 onward has interrupted transmission of the parasite (Higazi et al., 2013, Zarroug et al., 2014).

The proof-of-concept in Mali and Senegal and the decrease in infection prevalence in APOC countries resulted in expansion of the objectives of APOC and the onchocerciasis endemic countries in Africa to include elimination of onchocerciasis in 80% of African countries by 2025 (African Programme for Onchocerciasis Control (APOC) 2012b). Elimination of onchocerciasis in selected African countries was included among the WHO 2015–2020 targets for elimination or eradication of neglected tropical diseases (World Health Organization, 2012a).

### 1.5 The conceptual and operational framework of onchocerciasis elimination with ivermectin treatment in Africa

**Fig. 1:**
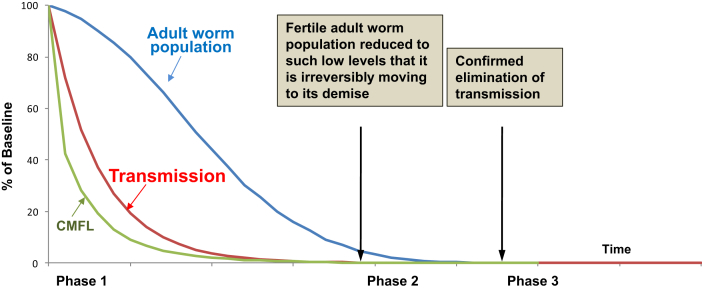
Conceptual framework for onchocerciasis elimination. CMFL - community microfilarial load. From: (African Programme for Onchocerciasis Control (APOC) 2010b).

Fig. 1 shows the framework for elimination of onchocerciasis developed by APOC (African Programme for Onchocerciasis Control (APOC) 2010b).

**Fig. 2:**
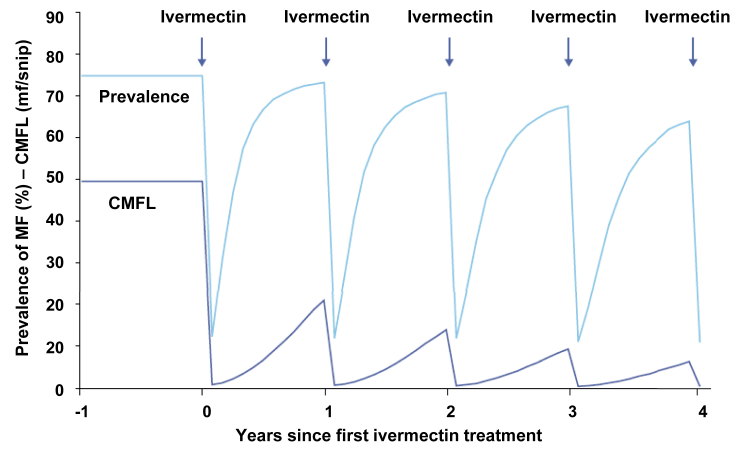
Simulation of the trend in prevalence after ivermectin treatment for different pre-control endemicity levels. The simulation was done with the programme ONCHOSIM (Plaisier et al., 1990, Remme et al., 1990). CMFL - community microfilarial load. MF - microfilariae. The geographic coverage and treatment coverage assumed are 100% and 70%, respectively. APOC recommends at least 80% therapeutic coverage for elimination of onchocerciasis. From: (African Programme for Onchocerciasis Control (APOC) 2010b).

In Phase 1, the intervention phase, implementation of annual CDTI leads to a progressive reduction of prevalence and intensity of infection in the population, usually quantitated via the ’Community Microfilariae Load’ (CMFL, the geometric mean of the number of microfilaria/snip of skin in a sample of >20 year old adults) (Remme et al., 1986). These reductions are the consequence of the killing of the vast majority of the skin microfilariae present in the human population within days of ivermectin treatment due to the ’microfilaricidal’ effect of ivermectin and the subsequent slow increase in skin microfilariae levels until the next treatment round (Fig. 2). The reduction in CMFL reduces the level of parasite transmission. Consequently, the macrofilariae population in the human population gradually decreases since the rate with which macrofilariae reach the end of their reproductive life span exceeds the rate of new infections reaching the adult life stage. When parasitological and entomological evaluations show that parasite levels in the human and vector populations have reached a level too low to sustain transmission across the whole transmission zone, CDTI can be discontinued. Phase 2 begins during which evaluations to verify that elimination has indeed been achieved are conducted. If elimination is confirmed, surveillance for timely detection of re-introduction of infection (or re-emergence in case of ’false negative’ conclusion from the verification of elimination) is initiated (Fig. 1).

New WHO guidelines for determining when treatment can be stopped and for verifying that transmission has been interrupted have become available. They include ’strong recommendations’ for testing the heads of black flies for *O. volvulus* DNA, i.e. for infective larvae (based on ’high certainty of evidence’) and testing of children under 10 years for presence of antibodies against the *O. volvulus* antigen Ov-16 (based on ’low certainty of evidence’). Only children are tested because the test detects patent as well as past infection. Consequently, other tests are needed to confirm patent infection in case of detection of antibodies to Ov-16 (World Health Organization, 2016).

**Fig. 3:**
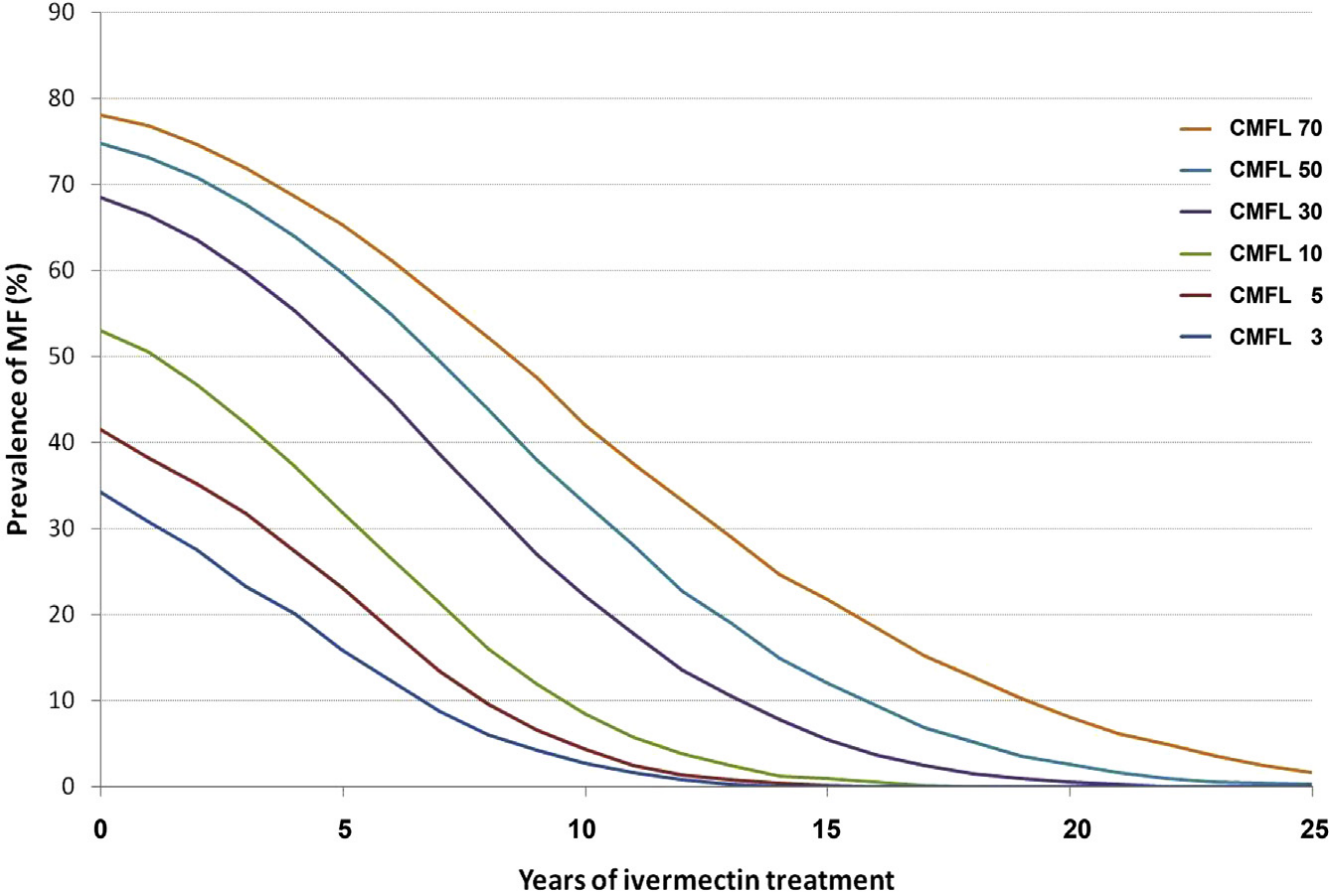
Trends in prevalence of people with detectable levels of skin microfilariae and community microfilarial load. CMFL - community microfilarial load. Mf - microfilariae. From: (African Programme for Onchocerciasis Control (APOC) 2010b). Prevalence refers to prevalence of people with levels of skin microfilariae detectable when two snips of skin are obtained and examined microscopically for presence of microfilariae, not to prevalence of infection.

The duration of the intervention phase depends on the CMFL before introduction of CDTI and the effectiveness of CDTI (Fig. 3). The pre-CDTI CMFL is a function of vector abundance and vector species since species differ with respect to their competency as vectors (Basanez et al., 2009). The effectiveness of CDTI depends on a number of factors including:

1. The vector species and abundance.
2. The percentage of the ’transmission zone’ in which CDTI is provided. A transmission zone is ’a geographical area where transmission of *O. volvulus* occurs by locally breeding vectors and which can be regarded as a natural ecological and epidemiological unit for interventions’ (African Programme for Onchocerciasis Control (APOC) 2010b).
3. The percentage of the eligible population which participates in CDTI (referred to as ’treatment coverage’) and the percentage of the population which never (or very rarely) takes ivermectin (referred to as ’systematic non-compliers’) (Turner et al., 2013, Coffeng et al., 2014a). The percentage of systematic non-compliers can be extensive. A study conducted among 8480 villagers from 101 villages in Cameroon and Nigeria after 8 years of CDTI showed that 5.9%, 8.5% and 12.3%, respectively had never, only once or only twice taken ivermectin (Brieger et al., 2011).
4. The susceptibility of *O. volvulus* to ivermectin and/or the level of immune competency of the host (see Section 1.6).

### 1.6 Challenges for elimination of onchocerciasis in Africa

The area over which onchocerciasis is endemic in Africa is huge. The latest update of the areas requiring interventions for elimination of onchocerciasis indicates that 172 Million people require treatment (African Programme for Onchocerciasis Control (APOC), 2015a, African Programme for Onchocerciasis Control (APOC), 2015b). A large number of endemic areas extend across country borders (Noma et al., 2014), including countries with different health care system capacity and history and risk of conflict. Both within and across countries, the start of CDTI implementation, the time at which full geographic and high treatment coverage were achieved as well as the extent of therapeutic coverage differ between geographic areas which may belong to the same transmission zone. Large areas are loiasis co-endemic (Zoure et al., 2011). Loiasis co-endemicity prohibits implementation of CDTI in onchocerciasis hypoendemic areas. The modified CDTI implementation strategy in onchocerciasis meso- or hyperendemic areas allows only a slow scale up to 100% geographic coverage (Mectizan Expert Committee and APOC Technical Consultative Committee, 2004). Furthermore, severe adverse reactions to ivermectin in loiasis co-endemic areas understandably reduce the willingness of the population to participate in CDTI (Haselow et al., 2003). In a *Loa loa* co-endemic area where CDTI had been going on for 10–12 years and severe adverse reactions had occurred at the start of CDTI, only 18% of participants had taken ivermectin at least 7 times, while 40.4% had taken ivermectin only 1–3 times and 15.5% had never taken ivermectin (Wanji et al., 2015). Finally, higher than anticipated prevalence of infection in areas under long term CDTI in Ghana and Cameroon have raised concern about emergence of parasites with low susceptibility to the embryostatic effect of ivermectin (Awadzi et al., 2004a, Awadzi et al., 2004b, Osei-Atweneboana et al., 2007, Osei-Atweneboana et al., 2011, Churcher et al., 2009, Pion et al., 2011, Pion et al., 2013, Nana-Djeunga et al., 2014, Lamberton et al., 2015). The fact that the response to the embryostatic effect can only be evaluated via repeated snipping of skin and microscopic examination for the number of microfilariae emerged after 24 h of incubation in physiological saline solution (Awadzi et al., 2004b, Osei-Atweneboana et al., 2007, Pion et al., 2011) makes it difficult to evaluate whether the frequency of people harboring parasites with low susceptibility to the embryostatic effect of ivermectin is increasing. The role of immunocompetency, linked to fast repopulation of the skin with microfilariae observed in Sudan (Ali et al., 2002), has not yet been evaluated. Furthermore, ’suboptimal response’ to ivermectin has also been observed in CDTI naive areas (Bakajika et al., 2013).

Many of these challenges can be addressed by improving implementation of CDTI, ideally supported by the results of social research to optimize community participation and achieve the 100% geographic and 80% treatment coverage recommended by APOC for elimination of onchocerciasis with annual CDTI. Biannual CDTI as conducted or planned in some areas in Africa may address other challenges, such as the emergence of parasites with low susceptibility to the embryostatic effect of ivermectin and the need to ’harmonize’ progress towards elimination across the different geographic areas belonging to the same transmission zone.

Other challenges to achieve elimination of onchocerciasis in 80% of African endemic countries by 2025 require new drugs. These need to have treatment regimens and safety profiles suitable for MDA or large scale individualized treatment within the health care capacities of the endemic countries as well as appropriate efficacy profiles (Table 1). From their conception, onchocerciasis control programmes have worked closely with researchers to address the challenges the programmes faced at different stages (Remme, 2004).

### 1.7 The path from discovery to qualification of drugs for human use

**Fig. 4:**
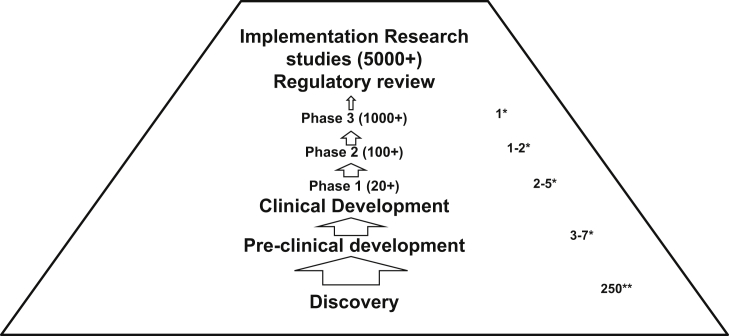
Stages, terminology and historical success rates during research of new drugs. **Discovery**: Testing of thousands of compounds in computer models, *in vitro* and in animal models with emphasis on evaluating whether the compounds have the desired efficacy. **Pre-clinical development**: Testing of compounds *in vitro* and in animal models with emphasis on evaluating the toxicity. On average around 5 of 250 compounds entering pre-clinical development are progressed to clinical development, i.e. are tested in humans. It was estimated that it takes 3–6 years to identify a compound that can enter clinical development. **Clinical development** consists of three phases. **Phase 1** studies occur typically in small groups of healthy volunteers. Out of 3–7 compounds tested in Phase 1 studies, 2–5 compounds enter Phase 2 studies. **Phase 2** studies are typically the first studies in people who have the disease intended to be treated. Out of 2–5 compounds tested in Phase 2 studies, 1–2 enter Phase 3 studies. **Phase 3** studies are typically conducted in ≥1000 people. Of 1–2 compounds tested in Phase 3 studies, one has the properties which result in submission for marketing approval by the US FDA. * Failure rates based on Congress of the United States, Congressional Budget Office, Research and Development in the Pharmaceutical Industry, October 2006 (http://www.cbo.gov/ftpdocs/76xx/doc7615/10-02-DrugR-D.pdf, accessed 16 January 2016) ** Failure rate based on estimate of International Federation of Pharmaceutical Manufacturer’s Association (IFPMA) presented to the WHO Expert Working Group on Funding R&D for Diseases of the Developing World (http://www.who.int/phi/IFPMA_FundingR&D_Jan09.ppt, access 16 January 2016).

Fig. 4 illustrates the different stages during research for new drugs to registration by a regulatory authority (i.e. approval of use in humans for a specified indication, formulation and dose regimen, also referred to as ’marketing authorization’) and qualification for large scale use in resource limited settings.

In contrast to discovery research, pre-clinical (non-clinical) development and clinical development need to be conducted according to guidance provided by regulatory agencies. The ’International Conference for Harmonisation on Technical Requirements for Registration of Pharmaceuticals for Human Use’ (ICH) was formed to harmonize regulatory requirements between the US, Europe and Japan. The ICH website includes the large number of guidance documents issued to date. ICH guidelines are not available for all testing required. Both the US regulatory agency (Food and Drug Administration (FDA)) and the European regulatory agency (European Medicines Agency (EMA)) give advice on drug development at request. Their websites include guidance on topics not covered by ICH guidelines. Table 3 provides websites and definition of some drug development terms.

#### 1.7.1 Pre-clinical (non-clinical) development

The objective of pre-clinical studies (also referred to as non-clinical studies) is to characterize the toxicity profile of a compound in terms of dose-response relationship, organs affected and reversibility of effects. A number of non-clinical studies required for registration may be conducted in parallel to clinical development. Guidance on what studies are required before initiation of clinical development is available. As clinical development proceeds, longer dosing schedules are evaluated and more participants are enrolled (see Fig. 4), additional non-clinical studies are required (e.g. ICH Guideline M3).

Pre-clinical studies include safety pharmacology and pharmacodynamic studies, toxicokinetic and pharmacokinetic studies, single and repeated dose studies to determine acute, subchronic and chronic toxicity, reproductive toxicity studies and genotoxicity studies. Carcinogenicity studies are required for compounds intended to have long treatment regimens and may be required for other compounds. Juvenile toxicity studies need to be conducted for compounds for paediatric use. Other types of non-clinical studies are dependent on compound characteristics, indication or as indicated by the results of other pre-clinical studies. Toxicity studies are conducted *in vitro* and in several animal species (rodent and non-rodent) to identify the species that provides the most appropriate model for a particular compound. With the exception of mode of action studies, usually conducted during discovery research, pre-clinical studies need to be conducted according to ’Good Laboratory Practice’ to support registration.

#### 1.7.2 Clinical development

Clinical development typically proceeds in 3 phases, but ’mixed phase’ studies are becoming more frequent.

Phase 1 studies occur typically in small groups of healthy volunteers. They are initiated with single ascending dose studies followed by multiple ascending dose studies if the intended dosing regimen includes multiple doses. Phase 1 studies are designed to determine the safety and pharmacokinetics of doses that can be expected to be efficacious based on the data from Discovery and pre-clinical development and whether the compound causes any adverse reactions that would prohibit further development. Phase 1 studies are also conducted to evaluate the pharmacokinetics, efficacy and safety in particular subpopulations (e.g. children, people with impaired liver or kidney function). Phase 1 studies in subpopulations may be conducted in parallel to Phase 2 or 3 studies.

Phase 2 studies are the first studies in people who have the disease intended to be treated. They are conducted in medium sized groups to determine whether the compound is likely to have the intended therapeutic effect without unacceptable adverse reactions.

Phase 3 studies are typically conducted in ≥1000 people. They are designed to test the hypothesis that the compound is as or more efficacious than the compound used for comparison (usually the ’standard of care’) and does not result in a level of adverse reactions not acceptable in the target population.

It was estimated that it takes around 6–7 years from the start of clinical development to submission to regulatory authorities for marketing approval (Dimasi et al., 2003, Congress of the United States - Congressional Budget Office, 2006, Pharmaceutical Research and Manufacturers of America, 2007, International Federation of Pharmaceutical Manufacturers & Associations, 2009, Food and Drug Administration of the United States of America, 2012).

At the time of registration, the regulatory authorities may require commitment to conduct additional studies (post-marketing studies, Phase 4 studies).

#### 1.7.3 Implementation research

For drugs intended for MDA (i.e. large scale use without diagnosis and treatment supervision by health care personnel), large scale trials (community studies) involving thousands of people are conducted to obtain additional data on the safety, efficacy and/or effectiveness of the drugs (Remme et al., 1989, DeSole et al., 1989a, DeSole et al., 1989b, Horton et al., 2000). Interventions intended for MDA and for individualized use in resource-limited settings also require implementation research. The studies are designed to understand and identify how to overcome barriers to effective use of the intervention. Such research resulted in e.g. the CDTI strategy and expansion of the strategy for delivery of other health interventions. Implementation research also provided the basis for todays strategy of home management of malaria (Horstick et al., 2010, Ajayi et al., 2013, Brieger et al., 2015, Sommerfeld et al., 2015). An implementation research tool kit is available http://www.who.int/tdr/publications/topics/ir-toolkit/en/(accessed 16 January 2016).

### 1.8 Regulatory agency advice and incentives for development of drugs for neglected tropical diseases

Both EMA and FDA try to encourage development of drugs for neglected tropical diseases through different mechanisms (Cole, 2010). This includes special provisions for advice on the development plan and/or assistance for development of study protocols.

The EMA provides ’scientific opinions’ for medicines (drugs, vaccines) to prevent or treat diseases of major public health interest outside the European Union (EU) in co-operation with WHO according to Article 58 of EC regulation 726/2004. Submissions for a ’scientific opinion’ undergo EMA assessment of quality, safety and efficacy according to the standards applied to submissions for registration/marketing authorization in the EU but in collaboration with WHO and regulatory agencies from non-EU countries (Table 2).

Since 2007, the US is incentivising investments into drug development for neglected tropical diseases through ’priority review vouchers’ (PRV) for organisations which register a drug for a qualifying disease. The PRV is based on a proposal developed by Ridley and colleagues (Ridley et al., 2006) and has also been suggested for Europe (Ridley and Sanchez, 2010). The PRV guarantees an FDA decision on the submission for another drug within 6 months and can be sold. For drugs with high economic potential, a reduction of the normal submission review period from 10 or more months to 6 months can result in significant profit since it increases the duration of market exclusivity (i.e. time during which the originator can market the drug without competition from generic drug manufacturers) by the number of months that the review period is shortened. The effectiveness of this approach and the rules under which a PRV is awarded have been examined critically (Sonderholm, 2009, Robertson et al., 2012, Sachs-Barrable et al., 2014, Kesselheim et al., 2015). The ’case’ of miltefosine for treatment of visceral leishmaniasis has pointed attention to the fact that a PRV can be awarded to an organisation which has not itself invested significant funds in the development and for a drug which has already been registered in another country for the neglected disease indication. One important factor in the effectiveness of the PRV is its market value. This has increased over time with the last PRV sold for US $350 Million (see Table 2 for the website reference which also includes other information on the PRV).

## 2 Research for new drugs for onchocerciasis control and elimination

Table 4 provides an overview of ongoing research by type of drug and stage of research classified as per Fig. 4. Based on the definitions in the ICH guidelines, all of the drugs and drug regimens in Table 4 are ’investigational products’ (see Table 3 for definition). The probability that adverse reactions (see Table 3 for definition) are identified which prohibit further development, registration or use, is lower when drugs are investigated which have already been approved at other doses, in other formulations, in combinations and/or for other indications for human use than for drugs without any registration for human use. Similarly and because toxicity testing required to register drugs for veterinary use is nearly as extensive as the toxicity testing to register drugs for human use, compounds which have already been approved for veterinary use are less likely not to progress to clinical development than compounds not registered for veterinary use. Therefore, the overview of the drugs in development is presented by regulatory approval history.

### 2.1 Drugs approved for human use for other indications

#### 2.1.1 Clinical development of new combinations and treatment regimens of drugs currently used for control and elimination of onchocerciasis and lymphatic filariasis

The DOLF project (Death to Onchocerciasis and Lymphatic Filariasis, for website see Table 2) is funded by the Bill and Melinda Gates Foundation (BMGF). The DOLF objectives are (1) to optimize MDA for LF with currently approved drugs and (2) to evaluate the efficacy of new combinations of the three drugs currently used for the control of onchocerciasis and LF (ivermectin, albendazole, as well as, outside Africa, diethylcarbamazine (DEC)) for onchocerciasis and LF. DEC is not used in onchocerciasis endemic areas of Africa because it can cause severe Mazzotti reactions in people highly infected with *O. volvulus* (Bird, 1980, Francis et al., 1985, Fulford et al., 1987, Awadzi and Gilles, 1992).

Table 4 shows the studies ongoing to evaluate the efficacy of combination treatment regimens against *O. volvulus*. The rationale for the addition of albendazole to ivermectin is based on the results of prior clinical studies. These suggested an effect of albendazole on adult worm fertility (Cline et al., 1992, Awadzi et al., 1995, Awadzi et al., 2003) which might be larger with higher and repeated albendazole doses.

#### 2.1.2 Clinical development of antibiotics against the *O. volvulus* symbiont *Wolbachia*

The discovery of intracellular bacteria in filarial nematodes dates back to the 1970’s (Kozek and Marroquin, 1977) and research into the role of *Wolbachia* on filarial development and the effect of treatment with antibiotics to the 1990’s (Bosshardt et al., 1993, Genchi et al., 1998). In 1999, the Special Programme for Research and Training in Tropical Diseases (WHO/TDR) organized a meeting on ’Wolbachia as targets for filarial control’ (Taylor et al., 2000) to review the evidence from non-clinical studies, assess the potential utility of drugs targeting *Wolbachia* for the control of onchocerciasis and LF and recommend research priorities.

Since then, it has been shown that *Wolbachia* is important for viability and fertility of *O. volvulus* (Tamarozzi et al., 2011). The results of a clinical study showing that depletion of *Wolbachia* results in *O. volvulus* sterility in people treated for 6 weeks with 100 mg doxycycline (Hoerauf et al., 2000, Hoerauf et al., 2001) have contributed to the funding of a large anti-*Wolbachia* (A-WOL) research programme by the BMGF (Table 2). The objectives of the A-WOL consortium are to (1) evaluate marketed antibiotics in *O. volvulus* infected people to identify regimens which are efficacious and safe with a dosing regimen suitable for large scale use, and (2) to discover new anti-*Wolbachia* compounds and develop them as appropriate to registration. The rationale and progress of this project have been reviewed regularly (Taylor et al., 2014).

Numerous clinical studies in onchocerciasis (or LF) have been conducted to examine the effect of different dose regimens of doxycycline, rifampicin and azithromycin (Hoerauf et al., 2001, Hoerauf et al., 2003, Debrah et al., 2006, Richards et al., 2007, Specht et al., 2008, Hoerauf et al., 2008a, Hoerauf et al., 2009, Turner et al., 2010, Debrah et al., 2015, Abegunde et al., 2016). To date, doxycycline has shown the highest efficacy. A modelling-based analysis of the data from three clinical trials of different regimens of doxycycline (Hoerauf et al., 2003, Hoerauf et al., 2008b, Hoerauf et al., 2009) concluded that there is no significant difference in the maximal proportional reduction of adult female *O. volvulus* containing *Wolbachia* between the regimens (91%–94%) and that *Wolbachia* depletion results in a 70%–80% reduction in the life span of *O. volvulus* macrofilariae (Walker et al., 2015).

The long duration of doxycycline treatment (≥4 weeks) as well as the safety profile of doxycyline (Table 2) limit large scale use and motivate ongoing research into the efficacy of other antibiotics (Table 4).

The clinical studies also showed that antibiotics have no acute microfilaricidal effect and that the decrease in skin microfilariae levels is due to microfilariae dying as they reach the end of their natural lifespan. This suggests that antibiotics will have a safety profile suitable for use in *Loa loa* co-infected people. A study which included *Loa loa* co-infected participants was conducted. No severe adverse reactions of the type reported after ivermectin treatment of people with high level of infection with *Loa loa* were reported. However, *Loa loa* microfilaridermia in participants was <8000 mf/ml (Turner et al., 2010). Since *Loa loa* does not contain *Wolbachia* (Grobusch et al., 2003, Buttner et al., 2003), antibiotics are no potential treatment for *Loa loa* infection.

Development of resistance to antibiotics is now recognized as a global threat (http://www.who.int/drugresistance/DC_Antimicrobial_Resistance/en/, accessed 16 January 2016). Therefore, use of antibiotics for *O. volvulus* or other filarial infections should include assessing the potential emergence of resistance of bacteria and other human pathogens treated with tetracyclines to ensure that use for parasitic infections does not negatively impact the ability to effectively treat other infections.

#### 2.1.3 Flubendazole

Flubendazole is a benzimidazole approved for treatment of gastrointestinal nematodes in humans.

A clinical study conducted around 30 years ago in Mexico with an injectable formulation showed that flubendazole was very efficacious against *O. volvulus* macrofilariae (Dominguez-Vazquez et al., 1983). At the time, the study was not completed due to injection site inflammation.

The promise of flubendazole as a macrofilaricide and the opportunities novel drug formulation techniques offer for an oral formulation with high bioavailability was pointed out by Mackenzie and Geary (Mackenzie and Geary, 2011). In 2011, a partnership between Michigan State University in the US, McGill University in Canada and the Not-for-profit organisation ’Drugs for Neglected Diseases Initiative’ (DNDi) was formed to develop a suitable oral formulation of flubendazole and conduct the relevant pharmacodynamic, pharmacokinetic and toxicology studies. The work of the partnership was supported by a grant from the BMGF (Table 2). In 2012, Johnson&Johnson joined the initiative (Table 2). DNDi has since completed its involvement in the project with transfer of all data to Johnson&Johnson (for website reference see Table 2).

New oral formulations have been developed (Ceballos et al., 2009, Ceballos et al., 2014). An oral formulation with higher systemic bioavailability than the formulations used for treatment of intestinal nematodes requires additional preclinical toxicology and pharmacotoxicity studies in view of the fact that flubendazole interferes with microtubules and can thus have reproductive toxicity (Mackenzie and Geary, 2011). Results of the first pre-clinical toxicology studies conducted have been published (Longo et al., 2013, Longo et al., 2014, Tweats et al., 2015).

The assessment of the results of the pre-clinical toxicology studies will determine whether an oral formulation can be developed clinically for MDA, for ’selective MDA’ (defined here as MDA excluding subpopulations susceptible to specific toxicity e.g. women of reproductive age due to reproductive toxicity), or for ’diagnosis-supported MDA’ (defined here as MDA which specifically identifies subpopulations with contraindications (e.g. pregnant women)). A ’diagnosis-supported MDA’ approach, referred to as ’Test&Treat’ approach, is currently being evaluated for ivermectin treatment for onchocerciasis in loiasis co-endemic areas (D’Ambrosio et al., 2015).

The data available from animal models and humans do not suggest that flubendazole kills microfilariae. Therefore, flubendazole is a very promising candidate for MDA in *Loa loa* co-endemic areas. Even if its safety profile should require ‘selective MDA’ or ’diagnosis-supported MDA’, flubendazole would be a very valuable tool for control and elimination of onchocerciasis: treatment of even only the male adult population would significantly reduce the burden of infection and transmission and reduce the population whose infections would need to be addressed with more individualized approaches.

Flubendazole should also be evaluated for its efficacy against *Loa loa* itself. Loiasis is not only an impediment to onchocerciasis and LF elimination, but also a neglected disease that puts a health burden on those infected (Andy et al., 1981, Nutman et al., 1986, Lukiana et al., 2006, Boussinesq, 2006). No effective and safe treatment is available. Given that albendazole has some limited effect on *Loa loa* (Kamgno et al., 2016) and current data on the relative effect of albendazole and flubendazole on *O. volvulus*, flubendazole may have anti-*Loa loa* efficacy.

Information on whether and when an oral formulation of flubendazole has or is expected to enter clinical development is not publicly available.

### 2.2 Drugs with proven efficacy in animal helminth infections but not approved for human use

Drugs developed for veterinary use are the ’classical’ source of compounds for development for human helminth infections. Two drugs approved for veterinary use are currently in development for onchocerciasis.

#### 2.2.1 Moxidectin

Moxidectin is, as ivermectin, albendazole and mebendazole, registered for treatment of different types of nematodes in animals. Both ivermectin and moxidectin contain a macrocyclic lactone ring, but ivermectin is an avermectin, while moxidectin is a milbemycin (Prichard et al., 2012). Research into moxidectin’s potential to have a higher effect on *O. volvulus* viability and/or fertility than ivermectin was initiated by WHO/TDR in the late 1990’s (Tagboto and Townson, 1996). Five Phase 1 studies in healthy volunteers were conducted (Cotreau et al., 2003, Korth-Bradley et al., 2011, Korth-Bradley et al., 2012, Korth-Bradley et al., 2013, Korth-Bradley et al., 2014). A Phase 2 study in Ghana determined whether moxidectin was safe enough in *O. volvulus* infected people to be evaluated in a Phase 3 study and obtained initial efficacy data (Awadzi et al., 2014). A Phase 3 study in ≥12 year olds with ≥10 microfilariae/mg skin was conducted in areas in Ghana, Liberia and the Democratic Republic of the Congo where CDTI had not yet been implemented at the time of participant recruitment (Opoku et al., 2013, Kanza et al., 2013, Bakajika et al., 2013). In both, the Phase 2 and the Phase 3 study, moxidectin was more efficacious in reducing skin microfilariae levels than ivermectin. More importantly, moxidectin prevented repopulation of the skin with microfilariae more effectively than ivermectin: skin microfilariae levels 12 months after moxidectin treatment were similar to those one month after ivermectin treatment (Opoku et al., 2013). Modelling of the effect of annual mass administration of moxidectin suggested that annual MDA of moxidectin can reduce time to onchocerciasis elimination relative to annual CDTI to an extent comparable to that achieved by biannual CDTI (Turner et al., 2015). The Australian Not-for-Profit organisation ’Medicines Development for Global Health’ (MDGH) to whom WHO has licensed all data available to WHO, intends to register moxidectin for onchocerciasis. MDGH will also sponsor a study to determine a paediatric dose and a large community study. MDGH furthermore intends to develop moxidectin for LF and scabies.

MDGH received the funds for the work required to prepare registration of moxidectin for onchocerciasis with the US FDA from the Global Health Investment Fund (GHIF). The fact that the US FDA would award a PRV if moxidectin was registered played a role in the GHIF funding decision (Table 2).

#### 2.2.2 Emodepside

Emodepside is a cyclooctadepsipeptide registered in Europe and the US as a combination product with praziquantel for treatment of cats infected with or at risk of infection with intestinal nematodes (*Toxocara cati Toxascaris leonina*, *Ancylostoma tubaeforme*) and cestodes (adult *Dipulidium caninum, Taenia taeniaeformis, Echinococcus multilocularis*) and as a combination product with toltrazuril for treatment of dogs with demonstrated or suspected infection with nematodes (*Toxocara canis*, *Uncinaria stenocephala*, *Ancylostoma caninum*, *Trichuris vulpis*) and coccidia (*Isospora ohioensis*, *Isospora canis*). Emodepside acts at the neuromuscular junction and has a unique mechanism of action relative to other antifilarial drugs, which is not yet fully understood (Harder and von Samson-Himmelstjerna, 2002, Harder et al., 2003, Harder et al., 2005, Willson et al., 2003, von Samson-Himmelstjerna et al., 2005, Krucken et al., 2012, Epe and Kaminsky, 2013, Kulke et al., 2014, Crisford et al., 2015). Pre-clinical pharmacology studies in *in-vitro* and *in-vivo* models of human filarial infections, including onchocerciasis models, sponsored in the early 2000s by WHO/TDR showed promising data (WHO/TDR, 2005, Townson et al., 2005). These contributed to emodepside being proposed as a candidate for development for human use (Olliaro et al., 2011).

In 2014, DNDi and Bayer HealthCare completed a legal agreement for collaboration on development of emodepside (Table 2). Clinical development has been initiated with the first study in humans to determine the safety, tolerability and pharmacokinetics of emodepside in healthy male volunteers (Table 4).

### 2.3 Discovery of new compounds

#### 2.3.1 New anti-wolbachia drugs or drug combinations

The A-WOL consortium has developed a *Wolbachia* cell-based assay for screening different compound libraries for compounds with activity against *Wolbachia*. The approach and results from screening of compounds already approved or in development for human use has recently been reviewed (Johnston et al., 2014).

Relative to the success rates shown in Fig. 4, it is noteworthy that historically antibiotics progressing from discovery and pre-clinical research into clinical development have a higher success rate than many other types of drugs with around 17% registered for human use (Kola and Landis, 2004). Assuming that no commercial considerations will interfere in progressing compounds identified by the A-WOL consortium into pre-clinical and clinical development, the success rate for anti-*Wolbachia* drugs could be even higher.

#### 2.3.2 Other compounds with activity against O. volvulus

A partnership of different research centres is funded by the BMGF for discovery of compounds which are cidal to *O. volvulus* in pre-clinical models and can be transitioned to preclinical toxicology studies (Table 2).

## Figures and Tables

**Table 1:** Challenges for accelerating progress towards elimination of onchocerciasis in Africa that can be addressed through availability of drugs other than ivermectin.

Challenges	‘Efficacy profile’ of new treatments
• Achieving at least 80% treatment coverage across 100% of the geographic area of each transmission zone • Accelerating reduction in infection prevalence and transmission in areas of a transmission zone where CDTI implementation started late, was implemented with low treatment coverage or interrupted due to conflict • *O. volvulus* strains with low susceptibility to ivermectin	• Treatment regimen(s)/drug(s) with higher efficacy against *O. volvulus* than ivermectin
• Co-endemicity of Loiasis	• Drugs effective against *O. volvulus* without a cidal effect on *Loa loa* microfilariae • Drugs with a sterilizing or cidal effect on *Loa loa* macrofilariae

**Table 2:** Website references.

Priority review voucher	http://www.priorityreviewvoucher.org/, accessed 8 April 2016
Development of new regimens of ivermectin and albendazole	DOLF project (Death to Onchocerciasis and Lymphatic Filariasis) website: www.dolf.wustl.edu, accessed 10 January 2016
Discovery and development of antibiotics	A-WOL consortium: http://a-wol.com/ University of Bonn (clinical development of anti-*Wolbachia* antibiotics): www.microbiology-bonn.de/immip/science Doxycycline label: http://www.accessdata.fda.gov/scripts/cder/drugsatfda/index.cfm, accessed 8 April 2016
Development of flubendazole	Bill and Melinda Gates foundation funding: http://www.dndi.org/media-centre/press-releases/918-flubendazole.html, accessed 8 April 2016. Johnson&Johnson involvement: http://www.jnj.com/news/all/johnson-and-johnson-joins-public-and-private-partners-in-the-largest-coordinated-action-to-date-to-eliminate-or-control-neglected-tropical-diseases, accessed 8 April 2016. DNDi involvement: http://www.dndi.org/diseases-projects/portfolio/completed-projects/flubendazole-macrofilaricide/, accessed 8 April 2016.
Registration of moxidectin	Medicines Development for Global Health: http://www.medicinesdevelopment.com/, accessed 8 April 2016 Global Health Investment Fund: www.ghif.com, http://www.ghif.com/uncategorized/press-release-moxidectin/, www.who.int/tdr/news/2015/moxi-treatmt-funding/en/index.html; both accessed 8 April 2016.
Development of emodepside	Labeling of emodepside containing products for veterinary use: http://www.ema.europa.eu/ema/pages/includes/document/open_document.jsp?webContentId=WC500063849, accessed 8 April 2016; http://www.ema.europa.eu/docs/en_GB/document_library/EPAR_-_Product_Information/veterinary/002006/WC500106176.pdf, accessed 8 April 2016; www.fda.gov/downloads/animalveterinary/products/approvedanimaldrugproducts/foiadrugsummaries/ucm062332.pdf; accessed 8 April 2016. DNDi and Bayer Health care agreement on development of emodepside: http://www.dndi.org/diseases-projects/portfolio/emodepside/, accessed 15 January 2016.
Discovery of macrofilaricidal drug candidates	University of California, San Francisco; Anacor; Lindsey F. Kimball Research Institute, N.Y. Blood Center; San Francisco State University http://tintin.sfsu.edu/gatesAward.html, accessed 8 April 2016
Websites with guidance on development of drugs for veterinary use	US FDA: http://www.fda.gov/AnimalVeterinary/GuidanceComplianceEnforcement/GuidanceforIndustry/default.htm EMA: http://www.ema.europa.eu/ema/index.jsp?curl=pages/regulation/landing/veterinary_medicines_regulatory.jsp&mid=

**Table 3:** Drug development terms and websites with information on drugs and drug development.

International Conference on Harmonisation of Technical Requirements for Registration of Pharmaceuticals for Human Use (ICH) and ICH guidelines	http://www.ich.org/about/history.html. ICH ‘Quality guidelines’ (qualification of drug substance, drug product and drug manufacturing): http://www.ich.org/products/guidelines/quality/article/quality-guidelines.html ICH ‘Safety guidelines’ (pre-clinical development): http://www.ich.org/products/guidelines/safety/article/safety-guidelines.html ICH ‘Efficacy guidelines’ (clinical development): http://www.ich.org/products/guidelines/efficacy/article/efficacy-guidelines.html ICH ‘Multidisciplinary guidelines’ (e.g. Medical Dictionary for Regulatory Activities, guidance for nonclinical safety studies required to support the different stages of clinical development): http://www.ich.org/products/guidelines/multidisciplinary/article/multidisciplinary-guidelines.html
US FDA	US Food and Drug Administration: http://www.fda.gov/ Guidances: http://www.fda.gov/Drugs/GuidanceComplianceRegulatoryInformation/Guidances/default.htm
EMA	European Medicines Agency: http://www.ema.europa.eu/ema/ Guidelines: http://www.ema.europa.eu/ema/index.jsp?curl=pages/regulation/general/general_content_000043.jsp&mid=WC0b01ac05800240cb Paediatric investigation plans: http://www.ema.europa.eu/ema/index.jsp?curl=pages/medicines/landing/pip_search.jsp&mid=WC0b01ac058001d129
Regulatory agency websites	US Food and Drug Administration: FDA, http://www.fda.gov/ European regulatory agency (European Medicines Agency, EMA): http://www.ema.europa.eu/ema/ EMA Scientific Opinions for medicines for use outside the European Union: http://www.ema.europa.eu/ema/index.jsp?curl=pages/regulation/general/general_content_000312.jsp&mid=WC0b01ac058001d12c
Public assessment reports, Summary basis of approval	Documents summarizing the assessment of the data submitted by the reviewers assigned by the regulatory authority. They include significantly more details than the label, prescribing information or package insert. European Public Assessment Reports: http://www.ema.europa.eu/ema/index.jsp?curl=pages/medicines/landing/epar_search.jsp&mid=WC0b01ac058001d124, Australian Public Assessment Reports: https://www.tga.gov.au/australian-public-assessment-reports-prescription-medicines-auspars US FDA for drugs: http://www.accessdata.fda.gov/scripts/cder/drugsatfda/index.cfm US FDA for biologics: http://www.fda.gov/BiologicsBloodVaccines/BloodBloodProducts/ApprovedProducts/LicensedProductsBLAs/
Product label, prescribing information, package insert	Documents summarizing information on the formulation, the results of pre-clinical development and clinical development, dosing and indications approved by the regulatory authorities. Agencies other than the ones listed below make labels publicly available on their websites. US Food and Drug Administration: http://labels.fda.gov/ European Medicines Agency: http://www.ema.europa.eu/ema/index.jsp?curl=pages/medicines/landing/epar_search.jsp&mid=WC0b01ac058001d124, Australian Therapeutic Goods Administration: https://www.tga.gov.au/australian-public-assessment-reports-prescription-medicines-auspars
Investigational drug	’A pharmaceutical form of an active ingredient or placebo being tested or used as a reference in a clinical trial, including a product with a marketing authorization when used or assembled (formulated or packaged) in a way different from the approved form, or when used for an unapproved indication, or when used to gain further information about an approved use.’, see e.g. ICH E6 Good Clinical Practice, http://www.ich.org/products/guidelines/efficacy/article/efficacy-guidelines.html, accessed 16 January 2016 Approved formulations and indications for the same active pharmaceutical ingredient (drug substance) may differ between countries.
Adverse event (AE)	Any untoward medical occurrence in a patient or clinical investigation subject administered a pharmaceutical product and which does not necessarily have to have a causal relationship with this treatment (ICH E6 Good Clinical Practice: http://www.ich.org/fileadmin/Public_Web_Site/ICH_Products/Guidelines/Efficacy/E6/E6_R1_Guideline.pdf)
Adverse drug reaction, adverse reaction (ADR)	For marketed medicinal products: A response to a drug which is noxious and unintended and which occurs at doses normally used in man for prophylaxis, diagnosis, or therapy of disease or for modification of physiological function. For new medicinal products or new uses of approved products: All noxious and unintended responses to a medicinal product related to any dose should be considered adverse drug reactions (ICH E6 Good Clinical Practice)
Serious adverse event (SAE) and serious adverse drug reaction (SAR)	A serious adverse event (experience) or reaction is any untoward medical occurrence that at any dose: - results in death, - is life-threatening, *NOTE:* The term “life-threatening” in the definition of “serious” refers to an event in which the patient was at risk of death at the time of the event; it does not refer to an event which hypothetically might have caused death if it were more severe. - requires inpatient hospitalisation or prolongation of existing hospitalisation, - results in persistent or significant disability/incapacity, or - is a congenital anomaly/birth defect. Important medical events that may not be immediately life-threatening or result in death or hospitalisation but may jeopardise the patient or may require intervention to prevent one of the other outcomes listed in the definition above should also usually be considered serious. Note: The terms ’serious’ and ’severe’ are not synonymous. The term “severe” is often used to describe the intensity (severity) of a specific event (as in mild, moderate, or severe myocardial infarction); the event itself, however, may be of relatively minor medical significance (such as severe headache). This is not the same as “serious,” which is based on patient/event outcome or action criteria usually associated with events that pose a threat to a patient’s life or functioning. Seriousness (not severity) serves as a guide for defining regulatory reporting obligations. (ICH E6 Good Clinical Practice)

**Table 4:** Overview of ongoing research for new drugs for onchocerciasis control and elimination.

Type of investigational drug	**Development phase** • Investigational regimens tested (country, estimated completion date, clinical trial registry number)
**Approved with different dosing regimens for other indications in humans**	
• New combinations and treatment regimens of ivermectin and albendazole (i.e. drugs currently used for control and elimination of onchocerciasis and LF in Africa)	Community studies* • Annual vs. biannual MDA with 400 mg albendazole + ivermectin (Ivory Coast, Dec 2017, NCT02032043) • Annual vs. biannual MDA with albendazole 400 mg + ivermectin (Liberia, April 2017, NCT01905436) Phase 2/3 clinical development* • Annual vs. biannual treatment with ivermectin 200 μg/kg or 200 μg/kg ivermectin +800 mg albendazole (Ghana, April 2016, ISRCTN50035143) • Biannual treatment with ivermectin 200 μg/kg with 0 mg, 400 mg or 800 mg albendazole. (Ghana, June 2017, NCT02078024) * all studies also assess efficacy against soil transmitted helminths
• Antibiotics with activity against *Wolbachia* in *in-vivo* animal models	**Phase 2 clinical development** • Rifapentine 900 mg/d plus moxifloxacin 400 mg/d for 14 or for 7 days, doxycycline 200 mg/d for 4 weeks(Ghana, May 2017, ISRCTN43697583)
• New oral formulation of flubendazole with higher systemic bioavailability	**Preclinical development** Time of decision on progression to clinical development unknown
**Approved for use in animals**	
• Moxidectin	**Preparation of submission for regulatory review** Preparation of community studies and study to determine safe dose in paediatric population
• Emodepside	**Phase 1 clinical development** • First in Human single ascending dose safety, tolerability and pharmacokinetic study (United Kingdom, April 2016, NCT02661178)
**New chemical entities**	
• Anti-wolbachia compounds	**Discovery/pre-clinical development**
• Other compounds	**Discovery**

MDA: mass drug administration, investigational regimens: regimens not used in CDTI or with regulatory approval for use in onchocerciasis.
